# Evaluating the Reliability of Health Portals’ Nutrition and Supplementation Advice for Pregnant Women: A Comprehensive Review

**DOI:** 10.3390/nu16111739

**Published:** 2024-06-01

**Authors:** Magdalena Skowrońska, Michał Pawłowski, Robert Milewski

**Affiliations:** 1 Doctoral School, Medical University of Białystok, 15-276 Białystok, Poland; magdalena.skowronska@sd.umb.edu.pl; 2 Department of Biostatistics and Medical Informatics, Medical University of Białystok, 15-295 Białystok, Poland; michal.pawlowski@umb.edu.pl

**Keywords:** pregnancy, nutrition, supplementation, health portals, evidence-based medicine

## Abstract

This article evaluates the reliability and consistency of nutrition- and supplementation-related advice for pregnant women provided by ten selected health-related Internet portals. The portals were chosen based on their perceived reliability and prominence in Google searches, with representation from both English and Polish language sources. The evaluation criteria included the adherence of the presented information to official recommendations and its evidence-based character based on specific items representing dietary aspects important in pregnancy. While the overall reliability was deemed acceptable, significant variations existed both among the portals and specific evaluated items. Notably, HealthLine, Medline Plus, and NCEZ emerged as the most evidence-based, while WebMD and Medycyna Praktyczna were identified as less reliable. Despite a number of issues, the analysed portals remain valuable sources of nutritional information for pregnant women, offering user-friendly accessibility superior to alternatives such as social media on the one hand and scientific articles on the other. Improved consistency and attention to detail, especially in relation to vitamin intake and supplementation, would improve the overall quality of health portals.

## 1. Introduction

Pregnancy is a time in a woman’s life when taking care of her (and the baby’s) health becomes an exceptionally important issue. The responsibility for another human being often becomes overwhelming, while the fear of making mistakes makes pregnant women turn to specialist sources for reliable information. Such sources range from physicians and other health professionals to social media, with the notion of ‘professionality’ not necessarily aligning with the actual quality of a source. Internet health portals are one of these, and their accessibility is an obvious asset.

It cannot be overlooked that, nowadays, the Internet is the main source of medical information for the majority of people. Although General Practitioners and specialists are still considered the ultimate source, the phenomenon of Dr Google has largely made inroads into this once indisputable area of expertise. It should also be emphasized that a previous study performed by the authors [1] showed that medical professional largely lacks the necessary nutritional knowledge to be able to offer reliable and consistent advice in this area, also in relation to pregnancy. The quality of health-related information available on the Internet varies wildly, and although recent years have seen some progress in both the positioning of relatively reliable sources in browser searches and awareness of misinformation and harmful advice one can encounter on the Internet, it is still challenging for most people to devise strategies for coping with this issue [2], which is especially important in the case of life-changing events such as pregnancy.

Pregnant women, especially first-timers, are naturally very concerned about potential mistakes that may influence the baby’s well-being. This state of mind may lead to overreliance on external information and a general approach that focuses on seeking advice in many different areas of life. The ability to employ critical thinking is an obvious issue in such situations, and studies show that although over 90% of women peruse Internet-based information, a large proportion may still display either excessive gullibility or unhealthy scepticism when it comes to external sources of information [3,4,5]. Due to the presence of factors such as stress and uncertainty, this tendency may apply to pregnant women to an even larger extent. Ideally, any patient would be able to make use of open access articles in medical databases such as PubMed in looking for the most recent and reliable data. Obviously, such an expectation is completely unattainable, and even if it were, the language of scientific publications would be beyond the grasp of the average non-medical reader. Still, one can reasonably expect that pregnant women could be able to effectively use the easily available Internet health portals. It is also not too far-fetched to expect that sources of this kind should contain evidence-based, consistent data on the nutritional and supplementation-related aspects of pregnancy. As health portals occupy a relatively neutral position among Internet-based sources, i.e., they are neither sensationalist nor overly scientific, they could play a pivotal role in guiding women through the often-confusing period, provided that they contain reliable information presented in a clear and—ideally—concise manner [3,4,5].

Several studies have been conducted with the aim of reviewing the reliability of popular Internet-based sources in the area of medical and nutritional advice. Bernard et al. [6] concluded that online information concerning diabetes lacks the necessary consistency and accuracy. Another study [7] reached a similar conclusion in relation to nutritional advice in general, emphasizing the fact that its recipients might be easily misinformed. A study performed by Sacks et al. [8], which investigated whether online sources concerning nausea and vomiting in pregnancy are evidence-based, showed that although most of the available information is accurate, this, in part, depends on whether the particular source is aimed at more literate readers or not, with the former relying on evidence to a larger extent. A similar problem was emphasized by Storr et al. [9], who concluded that both the accuracy of data and the user-friendliness of the analysed Internet-based sources focused on nutrition during pregnancy are significant problems.

For the reasons specified above, the aim of this article is to review ten selected health-related Internet portals as to the reliability and consistency of their nutrition- and supplementation-related advice for pregnant women.

## 2. Materials and Methods

Ten leading Internet portals (seven in English and three in Polish) focused on health-related information and advice were chosen for the study. The specific portals were selected on the basis of their perceived reliability, assessed on the basis of the following criteria:

(1) Does the portal provide references to professional/scientific literature?
(2) Is the quality/reliability of the quoted sources high?
(3) Is the portal affiliated with renowned institutions?
(4) Is the level of coherence and cohesion of the presented data high?
(5) Does the portal rely on scientific evidence?
(6) Is the information presented in an intelligible, precise manner?
(7) Are the authors professional dieticians or medical doctors?

All of the analysed portals met at least 5 of these criteria, which may have differed slightly in cases of individual articles published by the same portal. Affiliations with renowned organizations included the Mayo Clinic or the John Hopkins Institute, with all of the portals being updated by professionals and semi-professionals—as opposed to journalism-based Internet websites. Furthermore, the quality of presentation of information was also taken into consideration, which had to be simple enough to be understood by the target group and displayed in a user-friendly manner, at the same time relying on precise numerical data as much as possible. In general, the portals included in the study comprise most of the available high-quality sources of their kind, easily found in Google, thus constituting a representative sample.

The health portals included in the study were as follows:

1. English-language portals: HealthLine, WebMD, NHS, Mayo Clinic, John Hopkins, https://health.gov/ (accessed on 18 March 2024), Medline Plus;
2. Polish-language portals: 1000dni.pl, Medycyna Praktyczna, NCEZ.

The information published on the portals was analysed in the period from January to March 2024. The specific items to be analysed have been selected based on their relevance in pregnancy. Prior to browsing the portals, the authors made a list of all the important nutrients to be looked for (reflected in the tables in Section 3). The authors then analysed all available information concerning the aforementioned items found on the portals. Thorough searches of the websites were performed, e.g., if a given item was not discussed on pregnancy-related webpages of a given portal, its non-pregnancy-related ones, which might contain the relevant information, were browsed. The absence of data concerning a specific item was noted only after an exhaustive search of the relevant health portal had been performed.

The data were collected in a spreadsheet, grouped according to the analysed items (nutrient/ingredient/product/etc.), and collated with data available on each of the ten health portals. The contents of the portals were then compared with official recommendations issued by the relevant institutions and/or data available in peer-reviewed scientific articles. The recommendations were collected from the following sources:

1. World Health Organization (WHO);
2. European Food Safety Authority (EFSA);
3. Food and Agriculture Organization (FAO);
4. National Academy of Medicine (NAM);
5. American College of Obstetricians and Gynecologists (ACOG);
6. The German Nutrition Society (DGE);
7. Nordic Council of Ministers;
8. United States Department of Agriculture (USDA);
9. Department of Health & Human Services (HHS);
10. Health Council of the Netherlands;
11. French Food Safety Agency (AFSSA);
12. Polish Society of Gynecologists and Obstetricians (PTGiP);
13. Scientific articles from the PubMed database.

Data from scientific articles were used only if the two organizations listed above did not provide the necessary norms. The authors made sure that only the most recent norms were used.

The main purpose of the analysis of data was to decide whether Internet health portals as a whole provide reliable advice. In addition, in order to determine which individual health portals provide the most reliable and evidence-based information, the authors devised a simplified methodology consisting of giving each of the portals one point for each piece of information consistent with recommendations (it had to include dosages) and subtracting one point for each item that contradicted the official recommendations. The points were then summed for each portal, which would make it possible to rank the portals according to reliability of data. However, as the nature of the presented data and the very general character of some of the recommendations were not conducive to a clear-cut point-based analysis, the authors decided only to indicate those health portals that scored highest and lowest without actually ranking them.

## 3. Results and Discussion

Treated as a whole, the analysed health portals provide information concerning the following aspects of nutrition:

- Fluid intake, including caffeine, is mentioned by all the portals.
- Macronutrients are mentioned by all the portals:
  - Proteins are mentioned by all the portals; 5 out of 10 mention only specific products without the recommended intake;
  - Fats are mentioned by all the portals; only 3 out of 10 provide the recommended intake;
  - Carbohydrates are mentioned by 8 out of 10 portals; only 2 out of 10 portals indicate the recommended demand.
- Micronutrients are mentioned by all the portals:
  - Iron, calcium, and iodine are mentioned by all the portals; however, 2 out of 10 portals do not provide any concrete recommendations;
  - Zinc and potassium are mentioned by 7 out of 10 portals;
  - Magnesium and selenium are mentioned by 6 out of 10 portals.
- Vitamins are mentioned by all the portals:
  - Folic acid, vitamin A, and vitamin D are mentioned by all the portals;
  - Choline and vitamin C are mentioned by 8 out of 10 portals;
  - Vitamin E is mentioned by 7 out of 10 portals;
  - B-complex vitamins are mentioned by only 6 out of 10 portals, mostly superficially;
  - Vitamin K is mentioned by only 4 out of 10 portals.
- Herbs are mentioned by 6 out of 10 portals.

In general, the quality of information provided by the analysed portals proved to be adequate. Compared to other studies that assessed the reliability of Internet-based medical and nutritional information, this result might be considered slightly more positive, which most probably stems from the fact that the present study focused only on reputable health portals, as opposed to Internet-based sources of information in general.

In addition, as the study focused on English- and Polish-language health portals, a similar review of sources of this type published in other languages could be useful. It would offer insight as to whether Internet-based sources of medical and dietary advice that claim reliability are indeed evidence-based at an acceptable level regardless of the particular region of the world they are based in.

### 3.1. Generally Accepted Views

Some products are consistently contraindicated in pregnant women, both in official recommendations and by the analysed health portals. These include addictive substances such as cigarettes and alcohol [10,11]. In this respect, information on all medical portals is in agreement with recommendations stating that these products should be completely avoided during pregnancy due to their obvious and well-documented health risks.

Another area of agreement is foodborne pathogens. The danger of microbial contamination pertains as much to pregnancy as it does to the rest of society; it is, therefore, necessary to choose food products with due care to minimise the risk of infection and avoid the associated negative health effects. Among the products most commonly advised against for this reason are raw milk and other dairy products, meat (especially deli meats), eggs, fish (e.g., sushi) and seafood, salads (especially deli salads), refrigerated pâté and meat spreads, sprouts, and soft cheeses (although, in Poland, cheeses are produced mostly from pasteurized milk). In general, health portals agree that raw/undercooked and/or unpasteurized products should not be consumed during pregnancy.

While caffeine has traditionally been a topic of disagreement as to its health-related benefits or harms, there is, in fact, a greater scientific consensus in the area than the controversy would suggest. Fortunately, the analysed portals reflect the consensus. The safety assessment of caffeine published by Health Canada in 2003 is one of the most often cited sources in the peer-reviewed literature [12], recommending the dose not associated with adverse effects in healthy pregnant women, i.e., 300 mg/day. The same dose has been adopted in the WHO recommendations [13]. The European Food Safety Authority issued a more conservative recommendation in 2021 which stated that regular consumption of up to 200 mg of caffeine a day by pregnant and breastfeeding women can be considered safe [14]. Nine out of the ten analysed portals adhere to the findings of either of the aforementioned sources, with most of them recommending the lower limit. The only exception is Medycyna Praktyczna, which recommends that pregnant women should avoid coffee altogether. It should also be noted that products other than coffee, such as black tea or chocolate, are also rich in caffeine, and their consumption may lead to an accidental increase in daily caffeine intake.

A common misconception regarding coffee is that the beverage is seen as a disposable simulant despite the existence of studies pointing to its health benefits. In this context, the fact that the analysed portals do not perpetuate the aforementioned belief, at the same time providing accurate intake recommendations, must be seen as a very positive trait. Providing maximum daily intake is especially important considering the fact that many pregnant women may find it difficult to refrain from drinking coffee altogether. Still, more detailed information on other products containing caffeine would provide a broader perspective on the topic and increase nutritional awareness among pregnant women, especially when considering that the unit of coffee consumption used by the portals is usually ‘a cup’. This may be unintentionally misleading unless a clear distinction between, e.g., espresso and back tea is made in terms of their caffeine content.

Adequate fluid intake is another key aspect of a pregnant woman’s diet. According to EFSA, the total water intake in pregnancy should be 2300 mL/day [15]. However, data concerning the recommended water intake varies [16], and the available information is limited [17]. Similarly to the aforementioned situation, i.e., coffee not being the only product contributing to caffeine intake, it must be remembered that in the case of fluid intake in general, popular drinks such as coffee, tea, juices, drinks, and milk—as well as fruit and vegetables—also contribute to the overall fluid balance. This needs to be remembered in light of the fact that advice concerning fluid-rich products usually focuses on their other beneficial properties, and thus, the overall fluid intake may be, in fact, exceeded.

Fluid intake seems to be the most basic aspect of nutrition in general and in pregnancy in particular. Yet, although “drink plenty of fluid” is indeed the advice consistently repeated on the analysed portals, fluid intake is not, in fact, subject to specific WHO recommendations. Granted, specific doses may not be as important in this case as a general emphasis on proper hydration; however, in the authors’ opinion, specific WHO norms could hardly be regarded as detrimental, offering a concrete point of reference. Fortunately, EFSA fills this gap. The recommendations concerning fluid and caffeine intake in pregnancy are presented in Table 1.

Most portals are also in agreement as to their advice against extreme (i.e., deficient, one-sided) diets in pregnancy. This is the correct approach, as pregnant women are a special group in which the need for most nutrients is significantly increased. To ensure the proper development of the foetus, the diet should be as varied as possible and rich in all kinds of vitamins and minerals, which is impossible in the case of the popular extreme dieting regimens, e.g., keto or paleo.

Another area of general agreement throughout the analysed portals is those specific foods that are universally described as excellent sources of nutrients. One such product is whole grains, presented as rich sources of proteins, carbohydrates, or iron, with the specific focus varying on different portals. Other widely recommended foods include legumes (e.g., beans or peas), cereal, rice, nuts, or pasta. It must be noted, though, that the analysed health portals are often inconsistent in their approach to data presentation. For instance, some of them focus on providing advice on the amounts, or portions, of various foods to be consumed during pregnancy. In contrast, others give examples of products to be consumed when mentioning a particular nutrient. Still other portals prioritize supplementation, as opposed to a balanced diet, whatever particular diet a woman decides to adhere to. In the latter case, most portals display a pronounced focus on precision, reflected in providing specific doses. A minor concern must be mentioned concerning USA-based portals—they tend to provide data in Imperial Units such as pounds or ounces, which is an obvious hindrance for non-American users.

### 3.2. Macronutrients

Due to the need to maintain maternal metabolism and tissue growth, and to support foetal growth and development, the demand for nutrients increases during pregnancy [18]. Inadequate intake or deficiencies of key macro- and micronutrients can, therefore, have a significant impact on pregnancy outcomes and neonatal health.

It is a well-established fact that protein demand increases during pregnancy. Although all of the analysed health portals agree, the recommended amounts of protein intake are provided in two different manners—if at all. Some of the portals quote the total amount of protein to be consumed per day, expressed in grams/day, while others provide information on the recommended amount of protein intake, expressed in grams per kilogram of body weight/day. Given the substantial individual differences in body weight among pregnant women, it may seem that the more adequate way to provide the recommended amount of dietary protein intake would be the latter, which would make it easier for pregnant women to estimate their individual protein needs, but this is largely a matter of personal preference. 

Half of the portals, i.e., Medline Plus, https://health.gov/ (accessed on 18 March 2024), John Hopkins, NHS, and WebMD, only report the need for an increased daily protein intake during pregnancy without giving any specific data. Medycyna Praktyczna, 1000dni.pl, and Mayo Clinic, on the other hand, report the range of recommended protein intake for pregnant women, i.e., from 54 to 96 g/day. Only NCEZ and HealthLine give recommendations for protein intake in terms of g/kg of body weight. Still, there is a slight difference between the data presented on the two portals, as HealthLine’s recommendation is 1.1 g/kg body weight, while NCEZ’s recommendation is 1.2 g/kg body weight.

Scientific recommendations in this area are also inconclusive. The recommendations on protein requirements in pregnant women issued in 2005 by the Institute of Medicine (IOM) state that the EAR (Estimated Average Requirement) in pregnancy is 0.66 g/kg bw/day in the first trimester. In the second and third trimesters, protein intake increases by an average of 21 g/day, resulting in an EAR of 0.88 g/kg bw/day. In contrast, the RDA (Dietary Reference Intake) is 0.8 g/kg bw/day in the first trimester and 1.1 g/kg bw/day in the second and third trimesters [19]. In 2001, EFSA identified protein needs of 1, 9, and 28 g/day in the respective trimesters of pregnancy [20]. The latest recommendations, issued in 2019 by D-A-CH (the nutrition societies of Germany, Austria, and Switzerland), set the requirements for protein intake at 0.8, 0.9, and 1.0 g/kg bw/day during the first, second, and third trimesters, respectively [21].

It is worth noting that recommendations concerning the consumption of dairy as a source of protein specifically often lack the crucial clarification concerning raw, undercooked, or unpasteurized products, a distinction which is nevertheless mentioned when, e.g., milk itself is discussed, on a different webpage of the same portal.

With regard to fat intake recommendations, there is no consensus among the portals. John Hopkins simply states that fats should be kept to a minimum. https://health.gov/ (accessed on 18 March 2024) recommends eating low-fat products, while the NHS recommends limiting fatty products. The WebMD recommends consuming fats at 30% or less of the daily requirement. The 1000dni.pl recommends consuming fats between 30% and 35% of the daily requirement, while the NCEZ gives recommendations in the form of recommended increases in the amount of fat, given in grams for a specific trimester, i.e., +8–14 g/day in the second trimester and +11–18 g/day in the third trimester. The other portals do not specify the recommended fat intake for pregnant women.

Official recommendations set by the WHO do not specify the total fat intake in pregnancy. EFSA, on the other hand, indicates that fat supply during pregnancy may comprise 35% of the total energy intake [22]. These data are also reflected in the information provided by WebMD and 1000dni.pl.

An important issue that needs to be addressed in the context of fats is omega-3 polyunsaturated fatty acids (PUFAs). In the context of the human body, the most important omega-3 PUFAs are those sourced from fatty fish, i.e., long-chain omega-3 (LCn3) acids, including eicosapentaenoic acid (EPA) and docosahexaenoic acid (DHA), and plants, i.e., alpha-linolenic acid (ALA) [23]. Health portals give fatty fish, nuts, seeds, or avocados as sources of healthy fats, which is the correct advice.

On the other hand, the analysed portals only mention EPA and DHA acids, omitting the topic of ALA acids altogether, even though they are also an omega-3 fatty acid. EPA and DHA acids are extremely important components in the context of pregnancy [24,25], and their only source in food are fatty fish and seafood. The John Hopkins portal lists nuts as a source of omega-3 acids, which contains a significant understatement, as nuts are a source of ALA acid only; they do not contain EPA or DHA acids.

Only the Polish portals provide the recommended intake of DHA. 1000dni.pl recommends consuming at least 200 mg of DHA per day, more if fish consumption is insufficient, and 1000 mg/day of DHA in case of increased risk of premature birth. Medycyna Praktyczna recommends consuming 600 mg/day from the 20th week of pregnancy and 1000 mg/day in case of increased risk of premature birth. On the other hand, NCEZ recommends consuming 200 mg/day of DHA and 1000 mg/day of DHA in case of increased risk of premature birth throughout the entire pregnancy.

In general, information concerning omega-3 acids presented by the analysed health portals largely lacks consistency. As mentioned, only the Polish portals provide specific dosages, whereas the English-language portals only offer generic information on the sources of omega-3 acids, with example products differing between portals. Moreover, Mayo Clinic and Medline Plus recommend omega-3 supplementation only in case of deficiency, which is not strictly accurate, as supplementation with EPA and DHA omega-3 fats is recommended in pregnancy regardless of possible deficiencies [24,25]. It is also worth noting that while the health portals make mention of fish oil, explicitly or implicitly, in relation to omega-3 supplementation, they fail to provide meaningful details, with recommendations ranging from inconclusive, through overly cautious, to very general, i.e., in the form of statements on the benefits of fish consumption. Moreover, the related topics of fish, omega-3 acids, and fats in general may be described from different points of view on different webpages of the same portal without an overreaching depiction of all the relevant nuances in relation to one another.

As far as fats are concerned, no comprehensive recommendations exist that would provide advice regarding overall fat intake during pregnancy. Most recommendations for pregnant women relate to the consumption of specific types of fats, such as trans fats or omega-3 fatty acids. Recommendations from 2007, developed through a collaboration between several scientific societies, state that fat consumption during pregnancy should, in general, align with recommendations for the general population, adding that pregnant women should aim to consume an additional 200 mg/day of DHA, e.g., in the form of oily sea fish once a week [26]. It is worth emphasizing that because fats demand does not increase significantly during pregnancy and is similar to that of adults, this may be the reason for the lack of dedicated recommendations for pregnant women—as the norms for the general population apply [26].

Other FAO recommendations from 2010 suggest consuming 300 mg/day of EPA+DHA, of which 200 mg/day should be DHA [27]. On the other hand, EFSA recommendations from 2010 advise an additional 100–200 mg/day of DHA and over 250 mg/day of EPA+DHA [28]. Additionally, the World Association of Perinatal Medicine recommends 200 mg/day of DHA [26]. Recommendations also state that consuming long-lived predatory fish, which contain contaminants such as methylmercury and/or organic toxins, should be avoided [18]. The consumption of plant-derived alpha-linolenic acid is not recommended because humans poorly convert the omega-3 fatty acid into EPA and are unable to convert EPA into DHA in sufficient amounts [18].

In addition to PUFAs, evidence-based recommendations concerning saturated fatty acids and trans fatty acids are an important issue. Unfortunately, none of the analysed health portals referred to the recommended intake of saturated and trans fatty acids during pregnancy. Most drug societies recommend limiting the intake of saturated fatty acids to 10% [29,30,31] and trans fatty acids to 1% of daily energy requirements or as low as possible [22,29,31]. These recommendations apply to adults generally—there are no specific recommendations for pregnant women.

Although the analysed health portals pay considerable attention to fish and seafood—not only in relation to omega-3 acids—the advice they give is often both inconsistent and impractical. Due to their high nutritional value—especially in terms of macronutrients, particularly polyunsaturated fatty acids, including omega-3 acids—the consumption of seafood is generally recommended. Considering this, the health portals generally recommend fish and seafood as products appropriate for pregnant women if not consumed raw or undercooked, yet precise advice on which species are safe or unsafe to consume varies. For example, the portals recommend avoiding products such as high-mercury seafood and fish, raw fish, or cold-smoked fish, which is consistent with the scientific consensus, but some portals discourage the consumption of all smoked fish, which is an overgeneralization.

Mercury content is a problematic issue as far as the analysed portals are concerned. Nine out of ten (all except John Hopkins) advise against consuming high-mercury seafood and fish, yet only one (Mayo Clinic) proposes a useful heuristic, i.e., the bigger and older the fish, the more mercury it may contain. Such a practical approach enables the consumer to actually distinguish between high- and low-mercury fish and seafood, which would otherwise remain a very unclear and impractical distinction. Moreover, two other portals (HealthLine and WebMD) provide examples of high-mercury fish but none of high-mercury seafood. When low-mercury fish and seafood are described specifically, more examples are given of both, but despite the general consensus, i.e., a positive recommendation for their consumption, the details do not always align. For example, while WebMD classifies farmed salmon as a high-mercury fish, 1000dni.pl places it in the low-mercury category. Similarly, while 1000dni.pl recommends shrimp, NCEZ advises pregnant women to avoid it.

In terms of carbohydrates, the majority of the analysed portals do not provide precise information regarding the proportion of carbohydrates that would cover the needs of pregnant women. An important consideration in this context is that carbohydrates are always complementary to fats and proteins. It does not seem reasonable, however, to expect most women to be aware of this fact. For this reason, including information about this relationship on health portals would certainly prove beneficial and practical. Regarding food sources, the portals are rather consistent, recommending that the intake of simple sugars and highly processed products should be limited.

As far as recommendations issued by scientific societies are concerned, no specific guidelines regarding carbohydrate intake among pregnant women are given. However, in the case of recommendations for the entire adult population, according to the Nordic Nutrition Recommendations, carbohydrates (including energy obtained from dietary fibre) should comprise 50 to 60% of the total energy intake, while the intake of refined, added sugars should not exceed 10 E% [30]. In addition, the Netherlands Health Council recommends an intake of 272 to 282 g of carbohydrates per day, which is equivalent to approx. 40 E% [32]. In contrast, the German–Austrian–Swiss recommendations (D-A-CH, 2008) specify the level of carbohydrate intake at no less than 50 E% [29].

It would be useful to include information on the percentage of energy that should be provided by carbohydrates in the relevant recommendations, as exemplified by the recommendations quoted above. To make it easier for pregnant women to follow the advice and to understand the principles of healthy nutrition, such information should be included on health portals even if carbohydrates were to be obtained in the form of dietary supplements.

Another very important aspect regarding carbohydrates is dietary fibre. Unfortunately, none of the analysed health portals provide the recommended amounts of fibre to be consumed in pregnancy. There are also no official recommendations regarding dietary fibre intake for pregnant women. However, most scientific societies agree that the fibre content in the diet of adult women should be around 25–35 g/day [19,30,31,33]. More concerning is the lack of WHO norms for pregnant women concerning macronutrients, including carbohydrates and fibre, as these are provided only for the general population. Such omissions are reflected in the data available on the analysed health portals, as their advice is often vague and sometimes inconsistent. In the case of fibre, any mention is largely absent from the portals. Recommendations concerning macronutrients in pregnancy are presented in Table 2.

### 3.3. Supplementation: Microelements and Herbs

The concept of supplementation is closely connected with the overreaching aim of pregnant women to focus on their health and do whatever is necessary to make sure the baby will thrive. For this reason, using dietary supplements is seen as a desirable activity—one that doubtlessly leads to the improvement of wellbeing. This often results in the following overgeneralization: any supplementation is beneficial. This reasoning is obviously fallacious, and even more so in the case of pregnant women. While suboptimal micronutrient intake during pregnancy can lead to maternal nutrient deficiencies, associated with poorer pregnancy outcomes and adverse foetal outcomes, such as poor foetal growth, preterm birth, decreased infant survival, and increased risk of chronic diseases later in life [34,35,36,37], supplementation during pregnancy must be careful and based on actual recommendations.

The analysed health portals pay much attention to micronutrient supplementation, with varying levels of accuracy and consistency. Interestingly, almost none of the micronutrients is subject to stringent norms or recommendations issued by WHO or other relevant organizations, with the notable exceptions of iron and calcium. The primary focus of research concerning micronutrient deficiencies during pregnancy is overwhelmingly on folate and vitamin D [38]. Other micronutrients that are studied relatively often are iron, iodine, and calcium. In contrast, deficiencies in zinc, fluorine, selenium, and magnesium are given comparatively less attention despite their substantial impact on health. However, scientific studies rectify this gap to a certain extent by providing some relevant data [13,38,39], reflected in recommendations presented on several of the analysed portals.

Iron is a micronutrient essential for the process of tissue respiration, red blood cell formation, DNA synthesis, and the regulation of immune system function. Iron deficiency leads to anaemia, immune disorders, poor concentration, and cardiac arrhythmias. During pregnancy, anaemia increases the risk of premature labour and low birth weight [40]. Calcium, on the other hand, is involved in neuromuscular conduction, blood clotting, and regulation of the heart and blood vessels. Calcium deficiency causes increased excitability of the body, tetany, and blood clotting disorders and may also lead to increased blood pressure [41,42]. As far as iron and calcium are concerned, it can be noted that the analysed health portals generally recommend doses that are slightly below the WHO norms. This could be explained by the fact that the recommendations emphasize the fact that supplementation is necessary mainly in instances of deficiencies. In this context, the conservative approach does seem relevant. Even those portals that do not provide exact doses for iron and calcium recommend their supplementation, with the exception of Medycyna Praktyczna, which advises against calcium supplementation.

In the cases of zinc and magnesium, the health portals offer three types of information. These include providing general advice to supplement the mineral or pointing out its increased demand during pregnancy (four portals), the same advice but with exact doses given (two portals), or no specific mention of the microelement in the context of pregnancy (four portals). Magnesium is involved in neuromuscular conduction, thermoregulation, blood pressure regulation, and bone mineral metabolism. Its deficiency in pregnant women most often manifests as muscle cramps [43]. All dosages given for magnesium on the analysed portals are consistent with recommendations—even if they do not match the recommended doses exactly. In the case of zinc, for which there are no clear recommendations, WebMD and Medline Plus do, nevertheless, give specifics, i.e., 11 mg/day (as part of prenatal supplements) and <34–40 mg/day, respectively. It is impossible, however, to ascertain the sources of such data and, thus, its reliability. In general, it can be concluded that the advice found on health portals for zinc and magnesium is inconsistent but, fortunately, not harmful.

In the case of potassium, four portals view its supplementation as beneficial; another four do not mention it at all, while the rest advise against potassium in pregnancy supplementation beyond normal needs. As no official recommendations for the mineral exist, it is difficult to find such imprecise and inconsistent information reliable. The situation is similar with selenium. Some sources [39] suggest that the dosage determined for the general population, i.e., 45–75 μg/day, applies to pregnant women as well, which is indeed reflected on HealthLine, WebMD, and Medline Plus, albeit with some level of tolerance. In addition, Medycyna Praktyczna mentions an increased demand for the element, in contrast with 1000dni.pl, which states otherwise. None of the other portals makes mention of selenium in the context of pregnancy.

Iodine is an element essential for proper thyroid function. An insufficient supply of iodine in a pregnant woman’s diet can lead to the formation of thyroid goitre in the mother and mental underdevelopment in the child [40,44]. Out of the analysed microelements, iodine is the one which is described the most accurately by the analysed portals, the majority of which (six out of ten) provide recommendations falling closely within the recommended dose range of <220 µg/day. Three other portals recommend iodine supplementation without providing specific doses, while John Hopkins advises pregnant women not to supplement iodine.

While it is true that scientific sources fail to provide consistent data concerning the demand and/or supplementation needs of microelements during pregnancy—as reflected in the imprecise data available on the portals—more concerning are the omissions of certain microelements as part of the advice and the lack of clarity concerning the sources used for some of the specific data. On the other hand, it is worth emphasizing that the former problem may be due to a certain amount of caution on the part of the content creators, who try to avoid inaccuracies and prefer to err on the side of caution. Still, although the information concerning microelements provided by the analysed portals can hardly be regarded as potentially harmful, more openness in cases when the scientific consensus is tentative or non-existent could be helpful.

A concerning aspect of pregnancy-related supplementation is advice concerning herbs. It is a common phenomenon, not only in the public perception but also among some medical practitioners, to perceive herbs as a safe alternative to pharmaceutical drugs [1]. This is obviously fallacious reasoning, as the differing potencies of substances are not a consequence of their origin (i.e., a plant or a laboratory). This leads pregnant women who wish to avoid medicines altogether to turn to a safer—in their estimation—alternative, i.e., herbs. Fortunately, the analysed health portals generally recommend caution when it comes to the consumption of herbs, yet some of them make the proviso that ‘safe’ herbs can be consumed, seemingly disregarding the fact that scientific evidence on the safety of herbs, especially concerning pregnancy, is extremely scarce. For example, Medycyna Praktyczna informs that herbal teas made from those herbs that are safe can be drunk during pregnancy. Yet, as concrete data on the safety of particular herbs may be unavailable, it is virtually impossible to apply this seemingly sound advice in practice.

One of the herbs that is commonly advised against is black cohosh (*Cimifuga racemose*) used in supplements to alleviate the symptoms of menopause [45]. Indeed, the plant has been known for its labour-inducing properties, increasing the risk of miscarriage [46]. A related plant, i.e., blue cohosh (*Caulophyllum thalictroides*), has similar properties, in addition to a number of potentially lethal effects such as perinatal stroke, acute myocardial infarction, profound congestive heart failure and shock, and severe multi-organ hypoxic injury [47]. Scientific data concerning other herbs are exceptionally limited, especially considering their potential practical applications. As far as certain popular herbs used in supplements and as spices are concerned—data concerning ginseng is conflicting [48], while goldenseal and yarrow have been studied as part of a single pre-clinical trial, with yarrow proven to cause reduced foetal and placental mass [49]. Mugwort (*Artemisia Vulgaris*) [50] has been the subject of a meta-analysis, yet the study’s methodology is not robust enough to consider the results reliable [50]. Another herb, gingko balboa, has been found to induce perinatal bleeding due to its antithrombotic properties [51]. Linseed oil, on the other hand, has not been found to possess harmful properties. Wormwood (*Artemisia absinthium*) has not been tested in any scientific studies.

Interestingly, ginger, which is an herb often ingested as a measure to counteract nausea and vomiting, has indeed been found to be a safe and effective anti-emetic remedy in scientific studies [52,53]. A study performed by Boltman-Binkowski [52] showed that pregnant women who consumed ginger did not exhibit a significantly higher incidence of congenital abnormalities compared to those who did not. It did not, however, investigate whether ginger is protective against congenital abnormalities. Despite the scientific evidence and the popularity of ginger as a spice, which indicates that pregnant women might also wish to consume it, only half of the analysed portals recommend its use, with a further one (Medline Plus) advising caution. In addition, WebMD provides a recommended dose, i.e., 350 mg four times a day. Such precise data, however, seem cherry-picked, as various studies tested differing doses, which are indeed roughly in the quoted area [53], but no single dose has been indicated as the most accurate. In general, the example of ginger reflects the general lack of consistency of recommendations as far as herbs are concerned.

In general, herbs and herbal supplements need much more attention from health portals than they currently receive. Generally speaking, the haphazard information the portals provide seems to reflect the lack of research concerning herbal supplementation during pregnancy. Obviously, prospective studies of this kind would raise numerous ethical concerns, yet due to the fact that herbs themselves (e.g., spices) and herbal supplements are consumed widely and without much concern for their safety on the part of their users, pregnancy-related retrospective studies would provide the much-needed insight into the problem. As of the time of the writing of this article, pregnant women cannot expect to be given any useful advice concerning the herbs.

Overall, considering the huge number of herbs available as both dietary supplements and spices, the lack of evidence on their impact on pregnancy is troubling, with only two portals (HealthLine and Mayo Clinic) specifically mentioning the lack of research in the area as the reason for vague or non-existent recommendations. Still, the fact that health portals adopt a mostly very cautious approach to the consumption of herbs during pregnancy must be seen as positive. Recommendations concerning the supplementation of micronutrients and herbs in pregnancy are presented in Table 3.

### 3.4. Vitamins

In terms of vitamin supplementation, few are subject to WHO or other organizations’ standards. The only two officially recommended vitamins for supplementation are folic acid and vitamin D. 

All the analysed portals’ recommendations follow the WHO guidelines [13]. This means that in the case of folic acid, supplementation with 400 µg/day is consistently recommended, starting before pregnancy. However, portals such as HealthLine, WebMD, and MedlinePlus recommend that pregnant women should take up to 600 µg/day of folic acid. This does not follow from the WHO recommendations; however, EFSA does indeed state that the Adequate Intake (AI) of folic acid for pregnant women is 600 µg/day [54]. The Polish portals, namely Medycyna Praktyczna, NCEZ, and 1000dni.pl, provide in their recommendations dosages that vary according to the risk of neural tube defects (NTDs). They recommend 800 µg/day of folic acid in the case of a moderate risk of neural tube defects and 4000–5000 µg/day in the case of a high risk. These data are supported by studies whose results showed that a dose of 4000 µg of supplemental folic acid significantly reduced the incidence of births with neural tube defects [55,56]. Although most studies do not support supplementation with such high doses of folic acid, 4000–5000 µg is recommended in Poland in cases of mothers with a history of pregnancies diagnosed with neural tube defects [57].

Probably the most unexpected problem occurs in the case of B complex vitamins. In spite of the fact that the norms for B complex vitamins are widely accepted and easy to find, no portals apart from WebMD use the data in their recommendations. The other portals only gave general recommendations to supplement them without providing specific data, i.e., dosages. In the case of foods rich in these vitamins, recommended for consumption in pregnancy, most health portals only specify a general pool of foods rich in B vitamins; only the aforementioned WebMD lists foods rich in specific B vitamins and gives values for B vitamins similar to the EFSA recommendations [54]. When taking into consideration the easy access to recommendations concerning the B vitamin complex, this situation is rather curious. In the case of English-language portals, this could be explained by their focus on ready-made OTC vitamin supplements available from pharmacies, i.e., a belief may be prevailing that the doses they contain are consistent with the recommended ones. However, all the analysed Polish-language portals also have the same issue, and as they do not focus on bulk supplementation, there must be additional causes for such an approach. Whatever the reason, the authors feel that such information should be updated with specific numbers based on the latest evidence and recommendations.

Choline is a nutrient essential for the proper functioning of the human body. Preclinical studies have shown that high choline intake during pregnancy or the perinatal period has neuroprotective effects on the foetus [58,59,60]. Yet, most health portals only provide general recommendations for choline intake. No recommended doses are given. In contrast, the NHS portal does not mention choline at all in the context of the diet of pregnant women. The only portal that provides recommendations for choline requirements in pregnant women is HealthLine, with the recommended intake quoted as 450–930 mg/day, compared to the 480 mg/day recommended by EFSA [54]. Its extra-diet supplementation is not advised. The dietary sources of choline recommended by the different health portals are consistent with each other.

Vitamin C is a nutrient that is widely distributed in food. Although its demand during pregnancy does increase, up to 105 mg/day [54], additional supplementation is not recommended. Yet, only two health portals, i.e., Medline Plus and 1000dni.pl, mention this fact. Another portal, WebMD, states that the demand for vitamin C is 80–85 mg/day for pregnant women, which agrees with the recommendations issued by EFSA concerning the level of Vitamin C in the diet that meets the daily requirements of half of the people in a typical healthy population, i.e., the average requirement (AR).

Vitamin A intake is a crucial issue as far as dangers in pregnancy are concerned. Health portals should mention the dangers of excessive vitamin A consumption and its effects on the development of foetal defects more frequently and unambiguously. The requirement has been set by EFSA at 700 µg RE/day [54], with additional supplementation not recommended except in areas of widespread vitamin A deficiency [13]. Only the WebMD portal provides accurate recommendations for vitamin A intake during pregnancy, consistent with the EFSA guidelines. Importantly, it also includes information that vitamin A supplementation is not recommended for pregnant women, as does HealthLine. NCEZ and Medline Plus also warn against excessive vitamin A intake, mentioning that it can cause foetal defects, as confirmed both by WHO and in scientific studies [61,62,63]. Other portals provide only general recommendations without specific data. However, a list of specific foods rich in vitamin A to be avoided or at least consumed in moderation, would be a very beneficial piece of information for pregnant women.

Vitamin D is one of the two vitamins widely recommended for supplementation during pregnancy. Compared to the clarity of advice in the case of folic acid, the situation with vitamin D is more complicated. The WHO recommends supplementation only in cases of confirmed deficiency, while the requirement set by EFSA is 600 IU/day (15 μg/day). Furthermore, the WHO recommends vitamin D supplementation for pregnant women at 200 IU/day (5 μg/day) in cases of vitamin D insufficiency [13]. Most portals, however, recommend vitamin D supplementation at 600 IU/day. NHS recommends vitamin D supplementation of 400 IU/d from September to March due to the reduced solar radiation during these months, which is associated with reduced vitamin D synthesis. The Polish portals, i.e., 1000dni.pl, Medycyna Praktyczna, and NCEZ recommend vitamin D supplementation at much higher doses of 1500–2000 IU/day, with the dose increased to 4000 IU/day in cases of obesity. Although much different from the international recommendations, the data provided by the Polish portals is, in fact, consistent with the recommendations of the Polish Society of Gynecologists and Obstetricians [64]. This wide discrepancy in recommendations between international and Polish bodies is rather puzzling, and it would be interesting to know the reasons, as they are unclear.

According to the WHO and EFSA, the requirement for vitamin E during pregnancy is 11 mg/day [13,54]. Only WebMD indicates the need for this ingredient, yet the recommended amount differs slightly from the official recommendations of 13 mg/day. Only three portals, i.e., HealthLine, Medline Plus, and 1000dni.pl, report that regular vitamin E supplementation is not recommended. The other portals only give general or vague recommendations.

In the case of vitamin K, the requirement for pregnant women set by EFSA is 70 μg/day [54], i.e., the same as for the general adult population. As for the analysed health portals, the information they provide on vitamin K is very general. WebMD, Mayo Clinic, and Johns Hopkins report that regular vitamin K supplementation is not recommended during pregnancy, while the others make very general recommendations or do not mention vitamin K in the context of pregnancy at all.

Due to the relatively large focus of the analysed portals on OTC multivitamin supplements, the authors treated them as a separate issue. Although the WHO only recommends multivitamin supplementation for pregnant women in middle-income countries [65], all English-language portals recommend supplementation with OTC multivitamin preparations. In contrast, two Polish portals, i.e., 1000dni.pl and NCEZ, advise against supplementation with such preparations, while Medycyna Praktyczna has no clear recommendations on this issue. In general, it can be concluded that this issue has more in common with the easy availability of pre-prepared supplements in pharmacies than with their actual contents and their consistency with recommendations, with the analysed portals acting on the principle that officially approved products must be relatively well-prepared. This heuristic does not seem unreasonable, yet the authors feel that such ready-made solutions should be approached with a certain amount of caution, especially if they are not classified as medicinal products. Recommendations concerning the supplementation of vitamins in pregnancy are presented in Table 4. The data concerning multivitamin supplements, vitamins D and A, and folic acid concern the supplementation of these nutrients; in all the other cases, the data concern demand.

### 3.5. Pseudoscience

Pseudoscience can be defined as any claim, belief system, or practice that is presented as scientific, even though it lacks supporting evidence and plausibility [66,67]. This includes numerous popular practices commonly grouped under the umbrella term of ‘alternative medicine’, such as homeopathy or acupuncture, which either lack scientific evidence of their claimed benefits or are based on principles rooted in concepts based purely on belief and, thus, unfalsifiable/unprovable, e.g., astrology or faith-healing. Unfortunately, pseudoscientific content can be found on the analysed health portals, especially WebMD, which promotes a variety of unproven practices such as acupuncture, acupressure, chiropractic practices, and traditional Chinese medicine treatments, in particular, burning incense-like substance on the fifth toe. Acupressure (using bracelets or other methods) and acupuncture are also recommended by NCEZ. Acupuncture is endorsed by Mayo Clinic and HealthLine, which also promotes homeopathy. On the contrary, NHS correctly advises against all pseudoscientific practices, while the other five portals make no mention of them. Despite the fact that fewer than half of the studied portals display positive attitudes towards pseudoscience, it is a deeply disturbing phenomenon, especially in the case of the wide acceptance of acupuncture as a valid form of treatment [66]. Table 5 summarizes the presence of pseudoscientific content on the analysed portals.

It should also be mentioned that in the case of acupuncture and acupressure, the situation is blurred by the popular lack of understanding of the scientific consensus, i.e., the practices are not based on sound principles and lack evidence as to their efficacy [1]. Despite this, a case can be made as to their usefulness as relaxation practices. This implementation, however, and its positive results do not actually stem from the fallacious theoretical principles of the practices; they are rather an unintended consequence of the general massage-like character of acupuncture and acupressure. In other words, a pregnant woman may indeed find them relaxing and, thus, helpful, but if she does, this would not be the result of a proper application of the aforementioned unsound principles but of being subjected to the simple massage-like procedure. This distinction is not made sufficiently clear on the analysed health portals nor in other similar sources.

In the authors’ opinion, no pseudoscientific interventions or remedies should be mentioned on any health-related outlet, unless this is performed for the specific purpose of advising against such practices. This is one of the key principles of modern science [66,67] and one that needs to be strictly adhered to. In the case of the analysed portals, 4 out of 10 recommend pseudoscientific methods, with the two practices mentioned the most often being acupuncture and acupressure. Although the fact that pseudoscience is present in the minority of the portals, and the recommended interventions are relatively harmless, may be seen as satisfactory, it needs to be reiterated that health portals should refrain from all recommendations that are not evidence-based. There are several reasons for such a rigid stance. First of all, including unproven therapies within scientific content gives them undeserved legitimacy. Moreover, this may be seen as yielding under the pressure of women who are not aware that some practices are not evidence-based and expect them to be mentioned. In any case, pseudoscientific content does not reflect favourably on the portal publishing it, with WebMD being especially prone to this error.

## 4. Conclusions

As far as the reliability of the information provided by the analysed health portals is concerned in terms of their adherence to official recommendations, it varies quite substantially among the portals as well as in relation to the individual nutritional items. Generally speaking, although it can be stated that the overall level of accuracy is acceptable, there are still inconsistencies and areas that need improvement in light of the evidence-based provision of information and advice in order for the health portals to become fully reliable. It needs to be remembered, however, that such sources are not peer-reviewed and, thus, are not subject to rigorous content-editing processes. Moreover, many of their problems stem from the lack of scientific data or inconsistency of the research/evidence that they base their recommendations on.

The three health portals that have been found to be the most evidence-based and reliable are HealthLine, Medline Plus, and NCEZ. On the other hand, the two least evidence-based and reliable ones are WebMD and Medycyna Praktyczna. No discernible differences in quality were found between English- and Polish-language portals, even though they do differ in the particular aspects of pregnancy where they are likely to provide erroneous, insufficient, or inconsistent advice. In addition, it should be noted that the difference between the highest- and lowest-scoring portals was small enough to conclude that if a particular user prefers one portal over another due to certain secondary factors, e.g., the specific language used, presentation of information, website layout, interface, and their choice happens to be one of the lowest ranked portals, they are still unlikely to find extremely inaccurate or harmful data.

Considering that it is not typical behaviour on the part of pregnant women to seek advice in scientific databases, the analysed health portals provide a user-friendly and sufficiently reliable source of recommendations, much more useful and reliable than alternative options, e.g., social media or discussion forums.

## Figures and Tables

**Table 1:** Comparison between recommendations for pregnant women and information provided by the analysed portals concerning caffeine and fluid intake.

	Recommendations	HealthLine	WebMD	NHS	Mayo Clinic	John Hopkins	https://health.gov/ (accessed on 18 March 2024)	Medline Plus	1000dni.pl	Medycyna Praktyczna	NCEZ
Caffeine	<300 mg/day [13]	<200 mg/day	<300 mg/day	<200 mg/day	<200 mg/day	<200 mg/day	General recommendation to limit intake	<200 mg/day	300 mg/day	Caffeine to be avoided	200–300 mg/day
Fluid intake	2300 mL/day [15]	Recommended	Recommended	Not mentioned	Recommended	Recommended	Plain water recommended	Recommended	Recommended	1–2.5 L/day (still, mineral, low-sodium water)	>2 L/day (mainly still water)

**Table 2:** Comparison between recommendations for pregnant women and information provided by the analysed portals concerning selected macronutrients.

	Recommendations	HealthLine	WebMD	NHS	Mayo Clinic	John Hopkins	https://health.gov/ (accessed on 18 March 2024)	Medline Plus	1000dni.pl	Medycyna Praktyczna	NCEZ
Proteins	0.8 g/kg bw/day in the first trimester; 1.1 g/kg bw/day [19] Additional protein needs of 1, 9, and 28 g/day [20] 0.8, 0.9, and 1.0 g/kg bw/day [21]	Increased to 1.1 g/kg	General recommendation	General recommendation	71 g/day	General recommendation	General recommendation	General recommendation	54–96 mg/day	54–96 mg/day	1.2 g/kg bw
Fats	Up to 35 E% of total dietary intake [28]	General recommendation	Less than 30 E%	Limited consumption recommended	General recommendation	Limited consumption recommended	Low-fat foods recommended	General recommendation	30–35 E%	General recommendation	2nd trimester: 8–14 g/day; 3rd trimester: 11–18 g/day
Omega-3 fatty acids (DHA, EPA)	EPA + DHA: 300 mg/day [27] DHA: 100–200 mg/day; EPA + DHA: >250 mg/day [27] DHA: 200 mg/day [26]	General recommendation	General recommendation	General recommendation	Recommended if deficient	General recommendation	Not mentioned	Inconclusive	DHA: >200 mg/day; more if not enough fish consumed; if increased risk: 1000 mg/day	DHA: 600 mg/day from the 20th week of pregnancy; 1000 mg if risk of premature birth throughout pregnancy	DHA: 200 mg/day or 1000 mg/day; risk of premature birth throughout pregnancy
Carbohydrates	No specific information	General recommendation	General recommendation	General recommendation	Not mentioned	Keep to minimum	No specific data	General recommendation	45–65 E%	General recommendation	>175 g/day; 45–60 E%

**Table 3:** Comparison between recommendations for pregnant women and information provided by the analysed portals concerning supplementation of micronutrients and herbs.

	Recommendations	HealthLine	WebMD	NHS	Mayo Clinic	John Hopkins	https://health.gov/ (accessed on 18 March 2024)	Medline Plus	1000dni.pl	Medycyna Praktyczna	NCEZ
Iron	30–60 mg/day [13] (iron is not included in Polish recommendations)	27 mg/day	27 mg/day	General recommendation	27 mg/day	General recommendation	General recommendation	27 mg/day	<30 mg/day	General recommendation	General recommendation
Calcium	1500–2000 mg/day (in populations with low dietary calcium intake) [13,38]	General recommendation	1000–1300 mg/day	General recommendation	1000–1300 mg/day	1000–1300 mg/day	General recommendation	1000–1300 mg/day	1200 mg/day	No recommendation	General recommendation
Zinc	Supplementation only in case of deficiencies [13]	General recommendation	11 mg/day	No specific information	General recommendation	No specific information	No specific information	<34–40 mg/day	No recommendation	General recommendation	General recommendation
Magnesium	360–400 mg/day [38]	General recommendation	General recommendation	No specific information	No specific information	No specific information	Not mentioned	350–400 mg/day	200–1000 mg/day	Increased demand	Increased demand
Potassium	No specific information	General recommendation	General recommendation	No specific information	No specific information	General recommendation	General recommendation	No recommendation	No specific information	No specific information	No increased demand
Iodine	<220 µg/day [38]	220 μg/day	220 μg/day (safe limit: 1100 μg/day)	General recommendation	General recommendation	Recommendation to avoid	General recommendation	220 μg/day	150–200 μg/day	150 μg/day	150–200 μg/day
Selenium	45–75 μg/day (varies according to numerous factors; no specific recommendation for pregnant women) [39]	<70 μg/day	60–100 μg/day	No specific information	No specific information	No specific information	Not mentioned	60 μg/day	No extra supplementation needed	Increased demand	Not mentioned
Herbs	No specific information	Avoid (lack of research)	Small doses safe	If deficient (?)	Not enough scientific data; herbal tea not allowed	Not mentioned	Not mentioned	Caution recommended	Herbal tea allowed	Avoid	Not mentioned

**Table 4:** Comparison between recommendations for pregnant women and information provided by the analysed portals concerning vitamins.

	Recommendations	HealthLine	WebMD	NHS	Mayo Clinic	John Hopkins	https://health.gov/ (accessed on 18 March 2024)	Medline Plus	1000dni.pl	Medycyna Praktyczna	NCEZ
Folic acid	400 μg/day (supplementation; recommended start before pregnancy) [13] 600 μg DFE (dietary folate equivalents)/day (demand) [54]	400 (dose recommended for all women of childbearing age)–600 μg/day	400 (dose recommended for all women of childbearing age)–600 μg/day	>400 µg/day	400 μg/day (recommended start before pregnancy); 600–1000 μg/day	400 μg/day	400–800 μg/day (recommended start before pregnancy)	400 (dose recommended for all women of childbearing age)–600 μg/day	400–800 μg/day	400 μg/day	400 (dose recommended for all women of childbearing age)–800 μg/day
Vitamin BX	**B1**: 0.4 mg/1000 kcal (the same as for the whole population) [54] **B2**: 1.9 mg/day [54] **B3**: 6.6 mg/1000 kcal (the same as for the whole population) [54] **B5**: 5 mg/day (the same as for the whole population) [54] **B6**: 1.8 mg/day [54] **B7**: 40 μg/day (the same as for all adults) [54] **B12**: 4.5 μg/day [54]	General recommendation	Vitamins B1 and B2: 1.4 mg/day; vitamin B3: 18 mg/day; vitamin B12: 2.6 µg/day; vitamin B6: 1.9 mg/day (all doses based on prenatal vitamin supplements)	Vitamin B12 recommended for vegans/vegetarians; others not mentioned specifically	General recommendation	Sceptical toward vitamin supplementation in general	General recommendation	Vitamins B1 and B12 recommended; supplementation of the other B complex vitamins not advised	General recommendation	Increased demand	General recommendation
Vitamin B4 (choline)	480 mg/day [54]	450–930 mg/day	General recommendation	Not mentioned	General recommendation	General recommendation	General recommendation	Not mentioned	General recommendation	General recommendation	General recommendation
Vitamin C	AR: 80 mg/day PRI: 105 mg/day [54]	Recommended, but with some uncertainty	80–85 mg/day	General recommendation	General recommendation	General recommendation	Not mentioned specifically	Regular supplementation of vitamins not advised	Not recommended	General recommendation	General recommendation
Vitamin A	Only in situations where vitamin A deficiency is a widespread problem [13] 700 μg RE (retinol eq.)/day [54]	Supplementation not recommended	770 µg/day, supplementation not recommended	Not recommended	Supplementation of small doses recommended	General recommendation	General recommendation	Warning against possible birth defects	General recommendation	General recommendation	Warning against possible birth defects
Vitamin D	200 IU (5 μg)/day if deficient [13] 600 IU/day (15 μg) (the same as for all adults) (demand) [54]	600 IU/day	600 IU/day	400 IU (10 μg)/day between March and September; no more than 4000 IU/day	600 IU/day	600 IU/day	General recommendation	600 IU/day	1500–2000 IU/day, if BMI > 30-> 4000 IU/day	2000 IU/day	1500–2000 IU/day, BMI> 30–4000 IU/day
Vitamin E	11 mg/day [13,54]	Not recommended	15 mg/day	Not mentioned specifically	Recommended	Inconclusive	Recommended when taken with iron	Regular supplementation of vitamins not advised	Not recommended	General recommendation	General recommendation
Vitamin K	70 μg/day (the same as for all adults) [54]	General recommendation	Supplementation recommended only when needed	Not mentioned specifically	Not recommended	Sceptical toward vitamin supplementation in general	General recommendation	Inconclusive	Not mentioned	No increased demand	Not mentioned
Multivitamin supplements	Recommended for women in medium-income countries [65]	Recommended	Recommended	Recommended	Recommended as additional source	Yes	Yes (daily)	Yes	No recommendation	Inconclusive	No recommendation

**Table 5:** Pseudoscientific content mentioned on the analysed portals in relation to pregnancy.

	Recommendations	HealthLine	WebMD	NHS	Mayo Clinic	John Hopkins	https://health.gov/ (accessed on 18 March 2024)	Medline Plus	1000dni.pl	Medycyna Praktyczna	NCEZ
Pseudoscience	No mention whatsoever or strong advice against	Acupuncture, homeopathy	Acupuncture, acupressure, chiropractic practices, traditional Chinese medicine	Advice against pseudoscience	No mention	No mention	No mention	No mention	No mention	No mention	Acupuncture, acupressure
